# Modeling students’ instrumental (mis-) use of substances to enhance cognitive performance: Neuroenhancement in the light of job demands-resources theory

**DOI:** 10.1186/1751-0759-8-12

**Published:** 2014-05-26

**Authors:** Wanja Wolff, Ralf Brand, Franz Baumgarten, Johanna Lösel, Matthias Ziegler

**Affiliations:** 1 Department of Sport and Exercise Psychology, University Potsdam, Am Neuen Palais 10, 14469 Potsdam, Germany; 2 Psychological Institute, Humboldt University of Berlin, Unter den Linden 6, 10099 Berlin, Germany

**Keywords:** Neuroenhancement, Study demands, Burnout, Job demands resources theory, Students

## Abstract

**Background:**

Healthy university students have been shown to use psychoactive substances, expecting them to be functional means for enhancing their cognitive capacity, sometimes over and above an essentially proficient level. This behavior called Neuroenhancement (NE) has not yet been integrated into a behavioral theory that is able to predict performance. Job Demands Resources (JD-R) Theory for example assumes that strain (e.g. burnout) will occur and influence performance when job demands are high and job resources are limited at the same time. The aim of this study is to investigate whether or not university students’ self-reported NE can be integrated into JD-R Theory’s comprehensive approach to psychological health and performance.

**Methods:**

1,007 students (23.56 ± 3.83 years old, 637 female) participated in an online survey. Lifestyle drug, prescription drug, and illicit substance NE together with the complete set of JD-R variables (demands, burnout, resources, motivation, and performance) were measured. Path models were used in order to test our data’s fit to hypothesized main effects and interactions.

**Results:**

JD-R Theory could successfully be applied to describe the situation of university students. NE was mainly associated with the JD-R Theory’s health impairment process: Lifestyle drug NE (*p* < .05) as well as prescription drug NE (*p* < .001) is associated with higher burnout scores, and lifestyle drug NE aggravates the study demands-burnout interaction. In addition, prescription drug NE mitigates the protective influence of resources on burnout and on motivation.

**Conclusion:**

According to our results, the uninformed trying of NE (i.e., without medical supervision) might result in strain. Increased strain is related to decreased performance. From a public health perspective, intervention strategies should address these costs of non-supervised NE. With regard to future research we propose to model NE as a means to reach an end (i.e. performance enhancement) rather than a target behavior itself. This is necessary to provide a deeper understanding of the behavioral roots and consequences of the phenomenon.

## Background

Neuroenhancement (NE), the use of psychoactive substances to enhance one’s cognitive functioning, is prevalent [1-5]. In order to augment their already proficient cognitive capacity, healthy individuals use substances without medical instructions to do so [6]. Central to this substance-based account on NE are these substances’ biochemical functionality. However, actual substance functionality varies greatly between persons and situations [7,8], and substance-based definitions of behavior have been shown to be deficient in related domains, e.g. doping in sports [9]. In order to address this problem, a behavioral definition of NE has been recently introduced [10,11]. The assumed functionality of the consumed substance is central to this [10], and a similar perspective on instrumental drug use has been introduced, for example, to the related domain of doping in sports [12] and non-addictive drug use in general [13]: According to this, a student consuming a caffeinated drink (e.g. an "energy drink", *lifestyle drug NE*) as a means to increase concentration thus tries to neuroenhance. This outcome might be achieved even more effectively with amphetamine derivatives (e.g. Ritalin; *prescription drug NE*), or with an illicit substance (e.g. cocaine; *illicit substance NE*). But, by addressing the underlying behavior’s psychological roots (the individual consumes a substance as a means to reach an intended end; *means-to-end relation*) this opens alleys to investigate theoretically and empirically fruitful questions. For example, what psychological factors increase the probability of NE behavior and how will NE affect the individual’s psychological state in the long term (aside from the respective substance’s immediate and intended effects)? Throughout this article, NE will therefore be defined and measured as the medically unsupervised use of presumably psychoactive substances by healthy individuals who expect this substance to be a functional means of enhancing their cognitive capacity (sometimes over and above an essentially proficient level).

NE has primarily been studied in student populations. The lifetime prevalence of NE with coffee or caffeinated drinks for students has been reported to be 53% and 39%, respectively [14]. For prescription drug NE and illicit substances NE, the lifetime prevalence has been found to range from 7% to 9% among American students University and College; [15-17]. Research from other countries indicates that the NE prevalence might be subject to cultural variation [18,19]. For example the lifetime NE prevalence for Australian students is reported to be higher compared to their US or German counterparts [19]. One recent study employed a randomized response technique to assess the 12-month prevalence of NE among German university students [5]. These authors report a 20% prevalence for prescription and illicit drug NE. These considerable variations in reported NE prevalence are largely due to inconsistent classifications of substances (strict restriction to prescription and illicit substances in some studies [1,5,20], inclusion of different "softer," or more socially accepted substances, e.g. phytomedicine or energy drinks, in others; [3,10,21]). A recent review stresses further limitations of current NE research [22]: for example the unclear and differential effectiveness substances have on different cognitive functions. In our view, this provides a further call for the above proposed behavioral account on NE.

Most of the published social science (empirical) studies so far have focused on describing NE prevalence and its correlations with stressful demands [15-17]. Elaborations of the concrete settings’ psychological dynamics, in which associations between NE and demands and behavioral outcomes might occur, have been neglected so far. Examining this is at the core of our study.

Studies that directly assess the psychological and situational correlates of NE are scarce. The few exploratory studies are conducted in student populations e.g., [21]. Weyandt et al. [23] found higher global psychological distress to be associated with prescription drug NE in college students. Other authors argue that students take substances to deal with high study demands [3]. Further, one recent study found support for the hypothesis that being confronted with subjectively overwhelming demands is associated with higher rates of prescription drug NE in college students [10]. These studies indicate that different variants of NE differentially covary with situational (e.g. study demands) and psychological (e.g. mental health) variables.

In our view, there is a lack of theory-driven NE studies that imply the proposed means-to-end relation (i.e. consuming a substance as a means to improve cognitive performance). While epidemiological and exploratory research is a necessary first step in understanding a new phenomenon, theory-driven accounts constitute the necessary next step when a deeper understanding of a behavior is sought. Our main research goal is to provide a theoretical integration of NE behavior into one important psychological theory of student (employee) performance and health.

### The Job Demands-Resources Theory

**Figure 1:**
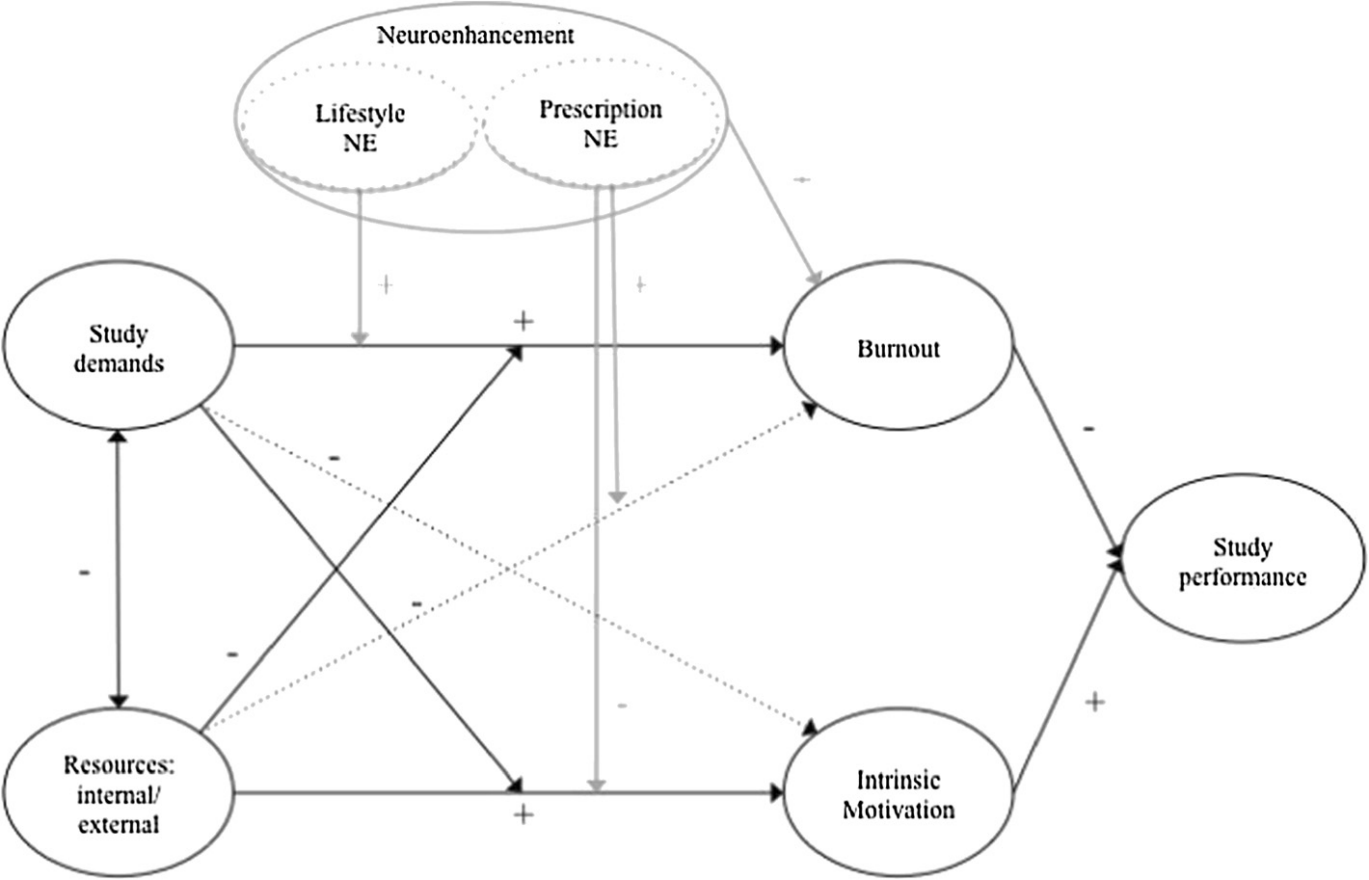
JD-R Theory and NE: the final model (Variables and effects of the general JD-R Theory are printed in black; Additional effects not predicted by JD-R Theory are printed in black interjected lines; NE and its effects are printed in grey).

Demands and mental health are associated with NE [10,23]. NE is used as a means to enhance performance among students [10,11]. Consequently, we turn to theories explaining the relationship of "workplace" factors with "employee" strain (e.g. mental health and burnout) and performance. Occupational theories have been successfully applied to student populations before [24-26]. Therefore, throughout this text our use of "occupation" and "work" comprises the educational setting as well. Job demands and job resources have both been associated with strain and motivation [27,28]. Most theories focus on the role of either job demands or of job resources [29]. Being one of the most comprehensive theories, Job Demands-Resources Theory JD-R Theory; [30] incorporates both of these variables. It proposes how demands and resources interact to predict motivation and strain as important psychological determinants of resulting performance (the full JD-R Theory is depicted in Figure 1).

### Demands

Demands are physiological, emotional, social and organizational conditions one physically or mentally has to deal with in the occupational setting [31]. High work pressure [31], time pressure, job complexity [32] and workload [33] are examples of such demands. Employees may use various performance protection strategies [34] to cope with them. Eventually chronic demands lead to depletion of mental and physical resources, which impairs both mental and physical health [35] as well as work performance [36]. This association between demands and strain is the first main effect specified by JD-R Theory. It has been labeled the *energetic process*[37] or *health impairment process*[31].

### Resources

Resources are defined as "those physical, psychological, social and organizational aspects of the job that are either/or: functional in achieving work goals, reduce job demands and the associated physiological as well as psychological costs and stimulate personal growth, learning and development" ( [31], p. 312). Resources can further be divided into (external) job resources and (internal) personal resources [29]. Job resources like decision latitude and social support originate from "outside" the person. Personal resources like self-efficacy originate from "within" the person see also, [38]. According to the job characteristics model [30], these resources are directly linked to work motivation. Hence, as a second main effect specified by JD-R Theory, resources are linked with motivational outcomes. This has been labeled the *motivational process*[37,39].

### Demands × resources

JD-R Theory postulates two interactions between demands and resources [31,40]. This allows for a wide range of different resources to moderate the demands-strain relationship [39]. Resources buffer the demand’s negative effects on strain (*buffer hypothesis*). Therefore, it is assumed that employees who have access to many job resources better cope with high demands [31]. As a second interaction, JD-R Theory proposes that demands moderate the association of resources with motivational outcomes. High demands elevate the positive effect of resources on motivational outcomes. This follows from the Conservation of Resources Theories’ COR; [41] claim that people aim for resource maintenance. Thus, imminent loss of resources with high demands fosters the motivational process [42].

### JD-R Theory: evidence and applications

In recent years, empirical support for JD-R Theory and the two proposed independent psychological processes (together with the respective proposed main effects) has accumulated e.g., [30,39]. Most of the empirical evidence supporting this theory stems from traditional occupational settings, while fewer studies were conducted within the educational context. For example, Bakker et al. [39] found that high demands were predictive for health related absenteeism in a sample of call center employees. In turn, resources (e.g. social support) were predictive of low turnover rates. Hakanen, Bakker and Schaufeli [43] found that burnout mediated the effect of high job demands on ill-health in a sample of Finnish teachers. Also, burnout mediated the effect of lacking resources on decreased work engagement. Salmela-Aro and Upadyaya applied JD-R Theory to the school context and found support for the theory’s postulated health impairment process [26]. In a sample of 1,709 Finish adolescent students, study demands predicted school burnout one year later. Further, school burnout negatively predicted school engagement (as a performance indicator), whereas depressive symptoms were positively related to school burnout over time.

Regarding the interaction of job demands and job resources, previous research is inconsistent [44]. Several studies indicate a moderating role of job resources on the health impairment process e.g., [42,45] and a moderating role of job demands on the motivational process e.g., [46,47]. Other studies do not find support for these effects e.g., [24,48].

To the best of our knowledge, JD-R Theory has so far not been applied to explain respective dynamics in university students. Empirical studies with a less complex forerunner of JD-R Theory, the Job-Demands Control (JD-C) Model, indicate that this is possible though [24,49,50]. Against this backdrop there are studies illustrating that university students sometimes struggle with high demands [51,52]. Motivation and academic success are known to be associated with external variables like control, autonomy and social support [24]. It is JD-R Theory’s flexibility in specifying the interplay of demands and resources on motivation, mental health and performance that leaves us confident in trying to adopt it to the university setting [29].

### Research questions

Our main research goal is to investigate how NE behavior, defined and measured as a means to enhance performance, can be integrated into a comprehensive theory of job-related health and performance. A crucial prerequisite for this is to test whether the JD-R Theory can be applied to the situation of university students. Within this theoretical framework, self-reported NE behavior is modeled as a moderator on JD-R Theory’s predictions.

NE has been found to be empirically associated with impaired mental health [23] and feeling overwhelmed from study demands [10]. This suggests that NE could be negatively associated with the health impairment process of JD-R Theory (demands - strain). Consequently, we expect NE to directly predict strain and to exert a moderating effect (aggravate) on the direct path between (high) demands and strain within JD-R Theory.

## Method

### Sample, setting, procedure

We identified 216 student associations from 71 German universities using a website that lists all German universities ( http://www.hochschulkompass.de) and approached them in order to distribute a link to an online questionnaire for their students via mailing lists (we do not know how many student associations actually distributed this link). In total, 2,229 students assessed the questionnaire during one month of data collection. Forty-five percent (*n* = 1,007) completed the questionnaire (mean age was 23.56 ± 3.83 years, 637 female).

On average, participating students had attended a university for 5.70 terms (i.e. almost three years) at the time of data collection. Participants reported spending a weekly average of 22.13 (±11.11) hours on lectures, 17.95 (±14.51) hours on private study and another 5.12 (±5.80) hours on other university related work. About half of them (48.50%) worked part time with the average being 12.20 (±17.60) hours. These values are representative for German university students [53]. Participation was voluntary and no compensation was offered for participation. Written informed consent was obtained by participants. Ethical approval for the study was granted by the ethics committee of the Department of Sport and Exercise Psychology at the University of Potsdam.

### Measures

#### JD-R Theory measures

Subjective work pressure was used to operationalize job demands [36]. These were assessed using a student-version [24] of the work pressure scale [54]. Sample items are: "There always seems to be an urgency about everything" and "It is very hard to keep up with your work load." Items were answered dichotomously with either "Yes" or "No." This scale’s internal consistency was α = .79 in our sample.

Burnout was used to operationalize strains [30]. Burnout was assessed using the Maslach Burnout Inventory-student survey MBI-SS; [55,56]. The MBI-SS is a 15-item questionnaire consisting of three subscales: exhaustion, cynicism and reduced efficacy that are combined for one overall burnout score. Sample items for the three scales included: "I feel emotionally drained by my studies" (exhaustion), "I doubt the significance of my studies" (cynicism), and "I can effectively solve the problems that arise in my studies" (reduced efficacy). Items were answered on a 7-point Likert-type scale with answers ranging from "Never" to "Always." Internal consistency for the overall scale in our sample was α = .89.

Self-efficacy was used to operationalize internal job resources [42]. It was assessed using the general self-efficacy scale [57] which consists of 10 items. Sample items included: "I can always manage to solve difficult problems if I try hard enough" and "I can usually handle whatever comes my way." Items were answered on a 4-point Likert-type scale with answers ranging from 1, "not at all true," to 4, "exactly true." The scale’s internal consistency in our sample was α = .84.

Decision latitude [40] and control [30] were used to operationalize external job resources. Decision latitude was assessed using the Decision Latitude Scale [58], which consists of nine items. To adapt the scale to students, the terms "work" and "job" were replaced by "studies." Sample items are: "My studies require that I learn new things" and "My studies allow me to make a lot of decisions on my own." Items have to be answered on a 4-point Likert-type scale with answers ranging from 1, "Not at all true," to 4, "Exactly true." Internal consistency in our sample was α = .68.

To assess students’ perceived control, we devised a five-item questionnaire. Items included: "The academic requirements of my university courses are transparent to me," "In my studies I have sufficient organizational freedom (e.g., selection of courses, studies abroad)," "In my studies I can influence the content of the academic curricula," "In my studies I can influence structural issues regarding my studies (e.g. study and examination regulations)" and "In my studies I can flexibly schedule my academic assignments." Items had to be answered on a 4-point Likert-type scale with answers ranging from 1, "Not at all true," to 4, "Exactly true." Internal consistency for the control scale in our sample was α = .65.

Intrinsic motivation was used to operationalize motivation [59]. It was assessed using the intrinsic motivation subscale of the academic motivation scale [60], consisting of 8-items. Items represent possible answers to the question "Why do you study?" Sample answers were: "…because I experience pleasure and satisfaction while learning new things," or "…for the pleasure that I experience when I feel completely absorbed by what certain authors have written." Items were answered on a 5-point Likert-type scale with answers ranging from 1, "Not at all," to 5, "Exactly." Internal consistency of the intrinsic motivation scale in our sample was α = .82.

Performance was assessed with a single-item self-assessment of academic performance: "How do you rate your study performance so far?" This item was answered on a 3-point Likert-type scale with answers ranging from 1, "I am probably in the lower third of my year," to 3, "I am probably in the upper third of my year." Additionally, the answer "I can not assess this" was provided.

#### NE behavior

Students were asked if they had ever tried NE ("Have you ever used a substance with the goal of increasing your cognitive performance") on separate questionnaire screens for lifestyle, prescription drug and illicit substance NE [10]. In addition, they were asked whether they currently used substances ("Do you currently use such substances?") on separate screens for lifestyle, prescription drug and illicit substance NE. In the descriptions, emphasis was placed on the means-end relation of NE use. This was necessary to help participants to not report, for example, their hedonistic cup of coffee during afternoon break.

#### Statistical analysis

All statistical analyses were conducted using the statistical packages SPSS 21 [61], R [62] and MPlus 7 [63]. Three different path models were used for hypothesis testing. The first model was used to test the prerequisite research question: Whether or not JD-R Theory can be fitted to university students’ data. The second and third models were used to test our main research question: The integration of NE into JD-R Theory. Model 1 represents the unmodified JD-R model. The manifest variables job demands, internal and external resources were correlated and used as predictors for strain and motivation. Additionally, after centering the variables, interaction terms were built and motivation and strain were also regressed onto the interaction terms. In a last step, performance was regressed on motivation and strain.

Model 2 starts with the same path model as described above. Additionally, current lifestyle drug NE use was used to predict strain and motivation. Moreover, the interactions with demands and both resources were also used as predictors of strain and motivation.

Model 3 is identical with Model 2 with the exception that current lifestyle drug NE was replaced with current prescription drug NE (analyses on illicit drug NE were not performed due to their low prevalence in our sample and the resulting lack of statistical power).

Models 2 and 3 thus explore the idea that different forms of NE might specifically affect different processes within the JD-R framework. In each model, all predictor variables were correlated.

Model tests were done according to the guidelines by Beauducel and Wittmann [64], Hu and Bentler [65] as well as Heene, Hilbert, Draxler, Ziegler & Buehner [66]. Thus, we looked at the global model test as well as the fit indices RMSEA (< .05), SRMR (< .08), and CFI (≥ .95). To correct for violations of multivariate normal distribution, a robust ML estimator was used. Missing data were estimated using the FIML method.

## Results

### Descriptive statistics

Descriptive statistics for all variables included in the model and for the NE variables are depicted in Tables 1 and 2, respectively.

**Table 1:** NE prevalence "Yes"

	**Prevalence**	
	**Lifetime**	**Point**
Lifestyle drug NE	83.20%	52.30%
Prescription drug NE	5.80%	3.00%
Illicit substance NE	3.50%	1.70%

**Table 2:** Descriptive statistics and correlations of JD-R model variables

				***r***											
	**Variable**	***M***	***SD***	**1**	**2**	**3**	**4**	**5**	**6**	**7**	**8**	**9**	**10**	**11**	**12**
1	Work pressure	0.73	0.26												
2	Self-efficacy	2.97	0.40	-.08											
3	Decision latitude A	2.12	0.63	-.35	.07										
4	Decision latitude T	2.90	0.38	.02	.19	.42									
5	Decision latitude overall	2.64	0.39	-.18	.16	.81	.87								
6	Control	2.17	0.53	-.38	.13	.61	.33	.54							
7	Burnout subscale-exhaustion	3.41	1.40	.46	-.28	-.21	-.05	-.14	-.29						
8	Burnout subscale-cynism	1.93	1.24	.09	-.25	-.16	-.32	-.30	-.20	.41					
9	Burnout subscale-ineffectivity	2.30	1.02	.19	-.38	-.15	-.20	-.21	-.20	.51	.54				
10	Burnout-overall	2.57	0.98	.33	-.38	-22	-.21	-.25	-.29	.83	.76	.84			
11	Intrinsic motivation	3.74	0.65	.12	.28	.09	.30	.24	.16	-.09	-.32	-.27	-.27		
12	Self-assessed performance	1.75	0.67	.17	-20	-.13	-.10	-.14	-.12	.22	.16	.40	.32	-.27	

*Note.* Correlations of .08 and above are significant at *p* < .001 (two tailed).

### Main analysis

Table 3 contains model fits for all three tested models. All models fit the data well. For each model, the standardized path coefficients are presented in Table 4. For Model 1 on the general JD-R Theory, strain as well as motivation were predicted by demands and both internal resources (all *p* < .001). The interactions between demands and internal or external resources predicted only strain. The impact was small but significant. The negative path weight shows that an increase in internal or external resources lowers the correlation between demand and strain (buffering effect). No such effect could be observed for motivation. All in all, 15.4% of the variance in motivation and 27.4% of the variance in strain could be explained. Strain (negatively) and motivation (positively) in turn affected performance (13.5% of explained variance). Thus, central JD-R Theory predictions could be applied to a student sample.

**Table 3:** Model fits

**Model**	**N**	***χ***^**2**^	**df**	**p**	**CFI**	**RMSEA**	**RMSEA low 90**	**RMSEA high 90**	**SRMR**
Model 1^a^	1005	17.356	5	.0039	0.979	0.05	0.025	0.076	0.017
Model 2^b^	1005	19.482	9	.0214	0.983	0.034	0.013	0.055	0.013
Model 3^c^	1005	20.733	9	.0139	0.981	0.036	0.015	0.057	0.016

*Note*. ^a^= JD-R Theory. ^b^= JD-R Theory including lifestyle NE. ^c^= JD-R Theory including prescription NE.

**Table 4:** Standardized path weights of all three tested models

	**Model 1**^**a**^		**Model 2**^**b**^		**Model 3**^**c**^	
	**Path weight**	***p***	**Path weight**	***p***	**Path weight**	***p***
**JD-R Theory**						
Main effects						
Demands on burnout	.270	< .001	.273	< .001	.262	< .001
Internal resources on motivation	.256	< .001	.252	< .001	.256	< .001
External resources on motivation	.248	< .001	.251	< .001	.246	< .001
Burnout on performance	-.266	< .001	-.267	< .001	-.266	< .001
Motivation on performance	.192	< .001	.191	< .001	.192	< .001
Interactions						
Demands * internal resources on motivation	-.016	.624	-.021	.515	-.006	.847
Demands * external resources on motivation	.051	.122	.053	.106	.054	.097
Demands * internal resources on burnout^d^	-.073	.008	-.079	.004	-.074	.007
Demands * external resources on burnout^d^	-.078	.009	-.075	.012	-.085	.005
Correlations						
Demands * internal resources	-.083	.010	-.083	.011	-.083	.010
Demands * external resources	-.294	< .001	-.294	< .001	-.294	< .001
Demands * motivation^d^	.205	< .001	.207	< .001	.204	< .001
Internal resources * burnout^d^	-.338	< .001	-.342	< .001	-.328	< .001
External resources * burnout^d^	-.178	< .001	-.174	< .001	-.185	< .001
**JD-R Model & NE**						
Interactions						
Demands * lifestyle NE on burnout			.050	.086		
Internal resources * prescription NE on motivation					-.092	.026
Internal resources * prescription NE on burnout					.076	.089
External resources * prescription NE on burnout					.045	.080
Correlations						
Lifestyle NE * burnout			.057	.032		
Prescription NE * burnout					.166	< .001

*Note.*^a^= general JD-R Theory. ^b^= JD-R Theory including lifestyle drug NE. ^c^= JD-R Theory including prescription NE. ^d^= effects that are not proposed by JD-R Theory but frequently found.

The main effects of demand and resources on motivation and strain were also observed in Model 2. The same holds true for the interactions between them. Lifestyle drug NE had a small but significant and positive effect on strain, i.e. a higher intake of lifestyle drug NE goes hand in hand with more self-reported strain. The interaction between lifestyle drug NE and demand marginally predicted strain. The positive weight shows that intake of lifestyle drug NE goes along with a stronger correlation between demand and strain. The amount of explained variance for both strain and motivation increased by 0.4% when adding lifestyle drug NE and the related interaction terms. The model’s predictive power for performance remained unaffected (13.5% of explained variance).

In Model 3, the JD-R Theory including prescription drug NEs is also built on the same main effects and interactions as observed in Model 1. Additionally, there are significant main effects of prescription drug NE on strain and motivation. The intake of prescription drug NEs goes along with more strain and less motivation. The interactions with internal and external resources are marginally significant predictors of strain. In both cases, a higher intake of prescription drug NEs increases the correlation between resources and strain. Moreover, there is a significant interaction between prescription drug NEs and internal resources predicting motivation. The negative path weight shows that a higher intake of prescription drug NEs decreases the correlation between internal resources and motivation. The amounts of explained variance increase to 16.4% for motivation and 29.4% for strain. Again, the model’s predictive power for performance remained unaffected (13.5% of explained variance).

## Discussion

This research assessed how NE interacts with the processes proposed by a comprehensive theory of job-related strain and performance. In line with our hypothesis, NE was associated with the JD-R Theory’s health impairment process. Lifestyle drug NE and prescription drug NE were significant predictors of higher burnout scores. Lifestyle drug NE further moderated the health impairment process. The negative influence of high demands on burnout was even higher in students using lifestyle drug NE. Prescription drug NE moderated the association of internal (self-efficacy) and external resources (decision latitude and control) with burnout scores. The positive influence of high resources on burnout was reduced in students trying prescription drug NE. In addition, prescription drug NE was associated with the motivational process. Prescription drug NE use moderated the association of internal resources with burnout. The positive influence of internal resources on motivation was reduced if students tried prescription drug NE. Differential predictive validity of lifestyle and prescription drug NE on model parameters accentuates the importance of a distinct assessment of NE variants. Finally, most central for the present study’s overall rationale, all of these variable relations concurrently predict study performance in the end.

According to JD-R Theory, study demands affect mental health by depleting the individual’s energetic resources [29]. This might explain the primary association of NE with the health impairment process. Our results indicate that students might try NE as fuel to counter detrimental effects of high study demands, and that such attempts might backfire. This interpretation can account for results found in previous explorative studies [3,10,23]. In line with the importance of stressful demands in student populations [24,49], this might help explain the possibly rising use of NE [5,15].

Whether or not NE is helpful at enhancing cognitive capacity might depend on situational and personal factors on the enhancing individuals’ side [7,8]. The general NE users’ current unaided reliance on lay knowledge might be a reason for the association of NE and burnout. We cannot rule out positive effects of NE in informed users (who may even be supervised by a physician). While this might be the case in the future, in the present most often it is not. Up until now, our results indicate that NE seems rather to be a deficiently employed means to achieve the goal of performance enhancement.

As a necessary prerequisite, we tested and found support for the hypothesis that JD-R Theory can be applied to university students. This finding expands the applicability of JD-R Theory and lends further support to the notion of university students’ work and traditional work sharing structural similarities [24]. When assessing single effects, both proposed main effects were evident in our sample: Supporting the prediction of a health impairment process, high study demands were associated with higher burnout scores. Supporting that of a motivational process, high external resources and internal resources predicted higher intrinsic motivation.

Internal and external resources moderated the study demands-burnout relationship. Higher resources mitigated detrimental effects of study demands on burnout. This supports JD-R Theory’s buffer hypothesis. No support for the moderating effect of study demands on the resources-motivation relationship was found however. This is not uncommon, as many studies fail to find any of these hypothesized interaction effects [48,67]. Beyond the effects proposed by JD-R Theory, we found a main effect of resources on burnout. High resources were associated with lower burnout scores. Albeit not explicitly postulated in the JD-R Theory, this effect has been reported in the respective literature e.g., [68].

JD-R Theory can be applied to university students. The prevalence of mental disorders in university students has increased [69,70] and college dropout numbers are on the rise [71]. Prevention and intervention programs are sought after [72]. The JD-R Theory can be used as a source for interventions [29]. Numerous respective recommendations from occupational settings could help to address similar issues for university students. Still, one has to keep in mind that this is the first application of the JD-R Theory to university students. Future studies should further investigate the JD-R Theory in this population and explore possible ways it needs to be modified to (even) better account for the situation of university students.

### Limitations

Although NE is significantly associated with burnout scores, one has to note that the amount of additional variance explained by this variable is rather small. In our view, this is not very surprising. One would simply not expect NE to explain vast amounts of variance in burnout scores given the multiple factors that are known to cause variation in this variable [73]. In line with this, one would not expect NE to play a much stronger role in moderating the effects of demands and resources on burnout. We think our results indicate the significance of NE among the multiple factors associated with burnout.

Our sample is self-selected and data collection was web-based. We cannot make inferences regarding the representativeness of our sample. However, our main goal was to achieve sufficient statistical power in order to test our hypotheses. NE is a socially sensitive behavior and self-reports might suffer from social desirability bias [5,74]. Consequently researchers have employed randomized response techniques RRT; [75] to arrive at valid prevalence estimates [5]. Although the NE prevalence in our sample is comparable to results of epidemiological studies, it is important to note that our study does not represent an epidemiological account on NE. Our research aims at deepening the theoretical understanding of how NE and psychological factors interact to predict performance (i.e. the end NE behavior is targeted at). RRT was not included in our study because this method is not informative on the individual level (i.e. it does not provide information on who uses NE and who does not). However, as NE prevalence rates from other studies [15,16] are comparable with the rate we observed in our study, we propose that this study’s sample is informative with regard to our research goal.

Our results are from a German sample and so far evidence suggests that NE might vary as a function of culture [19]. It is plausible to assume that the association of NE with the JD-R Theory variables is also subject to cultural variation. Personal and social factors have been linked to the hypothetical willingness to use NE substances [76]. Future research should therefore address cultural and other socially determined differences in actual NE behavior.

Our design is cross-sectional. Reversed causation cannot be ruled out. This limitation applies to both the general JD-R Theory and the integration of NE into JD-R Theory. NE could for example be the result of burnout. To the best of our knowledge, no longitudinal research on NE and only one experimental study on NE behavior have been published so far [11]. In order to arrive at conclusions concerning the temporal order of effects, longitudinal studies are needed. Cross-sectional evidence is a necessary prerequisite for prospective investigations of a phenomenon. To arrive at causal inferences, experiments are needed. Herein lies a most fruitful goal for future NE research.

## Conclusions

### Theoretical conclusions

This study is among the first to incorporate NE into a comprehensive theory of behavior. At least two theoretically important implications arise from this attempt. First, NE is understood as means-to-an-end within a behavioral pattern rather than as a behavioral outcome itself. This addresses what function corresponds to NE within JD-R Theory. Future research should address *why* individuals conceive NE as a functional means to achieve certain ends. For example, which ends (e.g., performance goals or health goals) contribute to conceiving of NE as a functional means? Second, NE’s behavioral core seems to be central in explaining the integration of NE into JD-R Theory. Different NE variants (i.e. lifestyle drug NE vs. prescription drug NE) differentially predict interactions that go beyond the general NE main effect. Resting upon a substance-independent behavioral core of NE, specific NE variants might add useful information with a more detailed embedding of NE into its respective means-to-end relation.

### Practical conclusions

In the future, NE might have a major impact on public health given its rising prevalence, the possible chances connected to it, as well as risks resulting from its uninformed use. This is important because an NE user might accept side effects of NE if the behavior is functional in achieving the intended ends. Our data indicate that side effects might come without the behavior leading to the intended ends. In fact, it may even make matters worse. For example a student might consume highly dosed caffeine in order to study longer hours. Her increased alertness might disturb general sleep patterns however, and cause exhaustion in the long run. Prevention strategies should address the ineffectiveness of the rather uninformed way students often utilize NE and emphasize that it might even be counter-productive, especially in light of under-estimated negative side effects, particularly of lifestyle drug NE [77,78]. We believe a JD-R Theory that incorporates NE might serve as a fruitful theoretical source for developing intervention strategies.

## Competing interests

The authors declare that they have no competing interests.

## Author’s contributions

RB directed the project. WW, RB, and FB designed the study. JL organized and performed the data collection. MZ and WW conducted the statistical calculations, WW wrote the first draft of the manuscript. All five authors then jointly worked on all subsequent versions of the manuscript. All five authors read and approved the final manuscript.
